# Procedure-specific pain trajectories and process quality in elective otolaryngology surgery: a single-center cohort study

**DOI:** 10.3389/fmed.2025.1682902

**Published:** 2025-11-14

**Authors:** Dandan Wu, Suqiong Ye, Yuee Zhang, Yuanyuan Zheng, Shuqin Qian, Nan Li

**Affiliations:** Department of Otorhinolaryngology, The First Affiliated Hospital of Guangzhou University of Chinese Medicine, Guangzhou, Guangdong, China

**Keywords:** otolaryngology, postoperative pain, opioid stewardship, enhanced recovery, China, quality of care

## Abstract

**Background:**

Procedure-specific postoperative pain in otolaryngology varies substantially, yet real-world associations between time-sensitive analgesic processes and patient-centered outcomes remain incompletely characterized in Chinese practice settings.

**Methods:**

This single-center prospective observational cohort (17 January 2021–31 March 2025) enrolled consecutive adults undergoing tonsillectomy, septoplasty, functional endoscopic sinus surgery (FESS), tympanoplasty/mastoidectomy, or microlaryngoscopy at a tertiary academic hospital in Guangzhou, China. Electronic health and anesthesia records captured 48-h worst pain [numeric rating scale (NRS) 0–10] and a composite process quality indicator [scheduled non-opioid ≤6 h plus timely post-anesthesia care unit (PACU) rescue ≤30 min for severe pain (NRS ≥ 7)]. Procedure category was the primary exposure. Worst pain was analyzed using ordinary least squares regression with restricted cubic splines and provider fixed effects; the process composite using logistic regression with heteroskedasticity-consistent standard errors.

**Results:**

Among 1,632 patients (mean age 38.5 years; 55.2% male), severe PACU pain occurred in 31.4%, rescue opioids in 42.5%, and timely rescue in 81.5% of those rescued (34.7% overall). Patients receiving scheduled non-opioids at discharge (80.3%) had lower 48-h pain (median 4.0 vs. 4.6), reduced opioid exposure (8.7 vs. 11.6 mg oral morphine equivalent), and higher satisfaction. Procedure category dominated pain outcomes: tonsillectomy versus FESS *β* = 1.14 [95% confidence interval (CI) 0.91–1.38]; microlaryngoscopy *β* = −2.02 (−2.34 to −1.70). For the process composite, higher opioid exposure predicted lower attainment (odds ratio per 5 mg 0.92; 95% CI 0.90–0.94), non-steroidal anti-inflammatory drug use predicted higher attainment (1.25; 1.00–1.57).

**Conclusion:**

Postoperative pain in ear, nose, and throat surgery is strongly procedure-dependent. Non-opioid-first regimens and timely rescue represent actionable quality improvement targets, requiring multicenter validation before broader implementation.

## 1 Introduction

Postoperative pain remains a pervasive and clinically consequential problem in otolaryngology, with important implications for recovery, unplanned health care contact, and patient-reported experience (1–3). Although many ear, nose, and throat (ENT) procedures are performed on an ambulatory basis, pain intensity in the immediate post-anesthesia period frequently reaches moderate-to-severe levels, and undertreatment in the post-anesthesia care unit (PACU) has been repeatedly documented (3, 4). These observations underscore the need for procedure-specific, process-aware analgesic strategies that reduce opioid exposure without compromising patient-centered outcomes (3–5).

Analgesic requirements differ substantially across common ENT procedures. Contemporary guidance for tonsillectomy recommends an analgesic foundation of paracetamol and non-steroidal anti-inflammatory drugs (NSAIDs), together with a single intraoperative dose of dexamethasone, reserving opioids for rescue; this reflects consistent evidence that tonsillectomy yields higher pain intensity than other ambulatory otolaryngology operations (5–8). Pain outcomes may vary by dissection technique (cold steel vs. electrocautery), though evidence on technique-specific pain trajectories remains mixed (3, 6, 7). In contrast, pain after septoplasty is typically mild and transient, while rhinoplasty produces moderate pain largely limited to the early postoperative period (3, 7, 9). Functional endoscopic sinus surgery (FESS) generally results in mild-to-moderate pain (typically NRS 3–5 on a 0–10 scale), and micro-laryngoscopy is often associated with lower pain intensity (NRS 2–4) compared to other ENT procedures, with postoperative throat discomfort related in part to suspension duration (5, 10). These procedure-stratified patterns underscore the need to benchmark outcomes and tailor care processes by operation type (5, 11).

The policy context further motivates opioid-sparing pathways. A comparative cohort study demonstrated markedly lower rates of postoperative opioid orders at a Hong Kong academic center than at a US institution across similar head-and-neck procedures, highlighting how multimodal non-opioid pathways and prescribing culture shape exposure (12, 13). Within China, multi-institutional and single-center surveys continue to report high rates of moderate-to-severe postoperative pain and heterogeneous practice patterns—features that signal substantial opportunity for standardization and stewardship (12, 14, 15).

Enhanced Recovery After Surgery (ERAS) frameworks and procedure-specific recommendations standardize perioperative care through multimodal non-opioid analgesia, antiemetic prophylaxis, and structured processes (16, 17). In otolaryngology, ERAS-style pathways are feasible and associated with fewer complications and shorter hospital stays, yet implementation fidelity and local effectiveness vary, underscoring the need for context-specific evaluation of process reliability and patient-centered outcomes (18, 19). Methodologically, two considerations are critical: postoperative pain follows distinct trajectories that single time-point measures obscure, and quality of acute pain management depends on both prescribed interventions and their timing, making “timely rescue” for severe pain a pragmatic quality indicator (20–23). Thus, optimal assessment should capture pain trajectories and process metrics reflecting early non-opioid scheduling and rapid escalation when pain is severe (19, 20).

Despite substantial postoperative pain burden and variability in analgesic practices reported in China, high-resolution, procedure-stratified evidence linking time-sensitive care processes to patient-centered outcomes remains scarce (24, 25). Few studies incorporate auditable process measures with 48-h outcomes, apply robust confounding control, or quantify how timely scheduled non-opioids and PACU rescue affect opioid exposure, pain trajectories, and satisfaction across common ENT procedures (3, 23, 26). This single-center prospective cohort has three objectives: (1) quantify procedure-specific pain trajectories (tonsillectomy, septoplasty, FESS, tympanoplasty/mastoidectomy, microlaryngoscopy as primary exposure); (2) evaluate a composite process quality indicator (early non-opioid scheduling ≤6 h plus timely PACU rescue ≤30 min for NRS ≥ 7); and (3) estimate adjusted associations between perioperative factors and worst 48-h pain (procedure category as primary exposure), assessing whether early non-opioid initiation reduces 0–24 h opioid exposure via inverse-probability weighting.

## 2 Methodology

### 2.1 Study design and setting

This was a single-center, prospective observational cohort conducted in the Otorhinolaryngology Department, The First Affiliated Hospital of Guangzhou University of Chinese Medicine (Guangzhou, Guangdong, China), spanning 17 January 2021 through 31 March 2025 (4.2 years). Consecutive eligible patients were enrolled prospectively at the time of surgery, with baseline and intraoperative data captured in real time via electronic health record (EHR) and anesthesia information management system documentation; postoperative pain assessments, medication administrations, and 24–48 h follow-up data were collected prospectively using routine clinical workflows. Data were subsequently extracted retrospectively from these prospectively completed records for analysis. Consecutive adults undergoing one of five common otolaryngology procedures—tonsillectomy, septoplasty, FESS, tympanoplasty/mastoidectomy, or micro-laryngoscopy—were included. The analytic cohort comprised 1,632 unique surgical cases, as reflected in the accompanying tables and figures. Care was delivered according to routine clinical practice; the team did not alter prescribing, administration, or discharge decisions. All measurements were derived from prospectively completed perioperative records and standard post-discharge follow-up.

### 2.2 Participants

Adults (≥18 years) undergoing elective ENT surgery under general anesthesia with planned PACU recovery were eligible if the index procedure was one of five prespecified categories—tonsillectomy, septoplasty, FESS, tympanoplasty/mastoidectomy, or micro-laryngoscopy—and core perioperative data were available to ascertain processes and outcomes. Required data included baseline pain [numeric rating scale (NRS) 0–10], PACU pain at arrival and discharge, time-stamped medication administrations to derive time to first rescue and total 0–24 h oral morphine equivalents (OME), and worst pain at 48 h obtained by routine telephone/app follow-up or documented contact. Exclusion criteria were age <18 years; emergency or add-on cases; procedures outside the five categories or combined/multisite operations not uniquely classifiable; concomitant major non-ENT procedures during the same anesthetic; missing primary outcome (worst pain at 48 h); absence of PACU pain documentation that precluded process assessment; and repeat operations within the accrual window (only the first eligible case per patient was retained). No exclusions were applied based on preoperative analgesic exposure, disposition, or language needs. Of 1847 consecutive patients screened during the accrual period, 215 were excluded: 18 age <18y, 31 emergency cases, 47 procedures outside five categories, 23 combined non-ENT procedures, 68 missing 48 h pain data, 19 missing PACU documentation, and nine repeat operations (first case retained), yielding 1,632 patients in the final analytic cohort (see flow diagram). Surgeons and anesthesiologists were captured as anonymized codes to enable provider adjustment without disclosure of personal information. Psychiatric conditions (including anxiety disorders, depression, or other psychological disturbances) were not exclusion criteria, as these comorbidities are common in surgical populations and their exclusion would limit generalizability; however, formal psychiatric diagnoses were not systematically captured from the EHR and thus could not be included as covariates in adjusted models. This represents an unmeasured confounder, as psychiatric conditions may independently influence pain perception, reporting, and analgesic requirements.

### 2.3 Data sources and variable definitions (measurement)

All variables were abstracted from the EHR, the anesthesia information management system, the medication administration record, and the routine 24–48-h telephone/app follow-up performed by the perioperative nursing team. Measurements and coding were standardized as follows:

#### 2.3.1 Sociodemographic characteristics

Age (years) was calculated from date of birth at the time of surgery; sex was recorded as male or female per the registration record. Marital status (single/married/divorced–widowed), education (primary or less/secondary/tertiary), employment (employed/unemployed/student/retired), residential setting (urban/peri-urban/rural), and insurance type (public/private/self-pay) were captured from admission intake forms. Household size (persons living in the patient’s household) and one-way travel time to hospital (minutes) were self-reported at preoperative assessment. Smartphone access (yes/no), interpreter requirement (yes/no), caregiver accompaniment on the day of surgery (yes/no), and hazardous alcohol use (yes/no per the institutional screening question) were documented by the preoperative nursing/anesthesia team. These sociodemographic variables were collected to characterize potential access barriers, social support, and social determinants of health that may confound associations between care processes and outcomes, following STROBE cohort reporting guidelines. Smartphone access was relevant for follow-up completion and digital health engagement; travel time and caregiver accompaniment were hypothesized to affect discharge planning and adherence; and household size was considered a proxy for social support. All data collection adhered to institutional privacy policies and national regulations, including the Personal Information Protection Law of the People’s Republic of China. De-identification and role-based access controls were implemented to minimize privacy risks.

#### 2.3.2 Behavioral and comorbidity variables

Smoking status was categorized as never, former, or current based on patient history. The American Society of Anesthesiologists (ASA) physical status was assigned by the attending anesthesiologist (I–III in this cohort). Diagnosed obstructive sleep apnea (OSA), diabetes mellitus, chronic kidney disease (CKD) or peptic ulcer disease (PUD), and asthma or chronic obstructive pulmonary disease (COPD) were abstracted from the problem list and preoperative history. Medication allergies to NSAIDs, acetaminophen, and opioids were coded as present/absent from the allergy registry.

#### 2.3.3 Preoperative analgesic exposure

Preoperative opioid status (naïve/intermittent/chronic) and regular preoperative non-opioid use (yes/no) were determined from medication reconciliation documented by the anesthesia team. These fields reflected clinical judgment integrating prescription history and patient report.

#### 2.3.4 Operative and anesthetic characteristics

Operative duration (minutes) was calculated from incision to closure times; estimated blood loss (milliliters) was taken from the operative note. Intraoperative opioid dose was converted to OME using the hospital’s conversion table and summed across all agents administered. Laterality (midline/unilateral/bilateral) and procedure specific descriptors were recorded as follows: tonsillectomy technique [cold steel dissection (intracapsular or extracapsular) vs. monopolar or bipolar electrocautery, as documented by the operating surgeon]; FESS extent [limited (uncinectomy, middle meatal antrostomy, anterior ethmoidectomy) vs. extensive (sphenoidotomy, frontal recess dissection, posterior ethmoidectomy, or multi-sinus involvement)]; tympanoplasty type (predominantly type I with fascia or cartilage graft; combined tympanomastoidectomy procedures were included if the primary reconstruction was type I tympanoplasty); and microlaryngoscopy laser use [CO₂ laser or potassium titanyl phosphate (KTP) laser was used in 62.4% (93/149) of microlaryngoscopy cases for vocal fold lesion excision, cordectomy, or laryngeal papilloma ablation; the remaining cases involved cold instrument biopsy or polyp excision without laser]. Use of the standardized ENT perioperative analgesia electronic order set (binary yes/no) was captured from the EHR. Local anesthetic infiltration at the surgical field (yes/no), maintenance technique [volatile-based vs. total intravenous anesthesia (TIVA)], airway device (endotracheal tube vs. laryngeal mask airway), and intraoperative adjuvants (acetaminophen, NSAID, dexamethasone, ketorolac, ketamine, dexmedetomidine; each yes/no) were abstracted from the anesthesia record. Antiemetic prophylaxis (yes/no) captured any prophylactic antiemetic administered intraoperatively.

#### 2.3.5 PACU processes and early postoperative outcomes

NRS pain scores (0–10) were recorded by nurses at PACU arrival and at discharge. Severe pain was defined as *a priori* as NRS ≥ 7. Administration of rescue opioid in PACU (yes/no) and the time to first rescue dose (minutes) were calculated from medication administration timestamps; when severe pain was documented, the interval was measured from the qualifying pain assessment to rescue administration. Timely rescue was defined as ≤30 min when pain was severe and ≤45 min otherwise. PACU length of stay (minutes) was calculated from PACU admission to discharge times. Postoperative nausea/vomiting (PONV) within 24 h (yes/no), presence of any sedation assessment documentation in PACU (yes/no), and disposition (ambulatory discharge vs. admission) were abstracted from nursing and discharge records. Total opioid consumption 0–24 h (OME, mg) included all opioids given intra- and postoperatively during the first 24 h, converted and summed using the institutional OME table.

However, rescue timeliness was assessed only among patients who received a rescue opioid dose in PACU. For episodes of severe pain (NRS ≥ 7), the interval was measured from the qualifying pain assessment to the rescue administration timestamp; for non-severe pain, the interval was measured from the immediately preceding pain assessment documenting the indication (or PACU admission time if no assessment preceded rescue). Patients without rescue had the timeliness metric coded as missing and were excluded from this measure.

The prespecified timeliness thresholds [≤30 min for severe pain (NRS ≥ 7) and ≤45 min for moderate pain] were selected *a priori* based on three considerations: (1) clinical guidelines emphasizing rapid escalation for severe acute postoperative pain to prevent suffering and adverse physiological sequelae; (2) prior PACU literature documenting that pain rescue intervals exceeding 30 min are associated with lower patient satisfaction and increased likelihood of inadequate analgesia; and (3) pragmatic feasibility in a tertiary PACU setting with routine nursing documentation cycles of 15–30 min. We acknowledge that a 30-min interval for severe pain management may be considered suboptimal by some quality standards, and that shorter thresholds (e.g., 15 or 20 min) may represent more aspirational benchmarks for high-acuity pain care. Our threshold reflects observed real-world practice constraints (including time for nursing assessment, physician notification, medication preparation, and administration) rather than an ideal standard. In our cohort, 81.5% of rescued patients met the 30-min threshold when pain was severe, suggesting that this represents an achievable but imperfect quality target. Sensitivity analyses using alternative thresholds (20 and 40 min) showed qualitatively similar patterns of composite attainment across procedure categories.

#### 2.3.6 Discharge prescribing and 48-h follow-up

At discharge, scheduled non-opioid analgesics (yes/no) and any opioid prescription (yes/no) were obtained from the electronic prescribing module. Follow-up at 24–48 h collected the worst pain at 48 h (NRS 0–10), adherence to scheduled non-opioids (yes/no among those prescribed), any unplanned pain-related health care contact within 72 h (yes/no, including emergency department visits, urgent clinic returns, or telephone escalation), and patient-reported satisfaction with pain management (0–10, higher is better).

**Figure 1:**
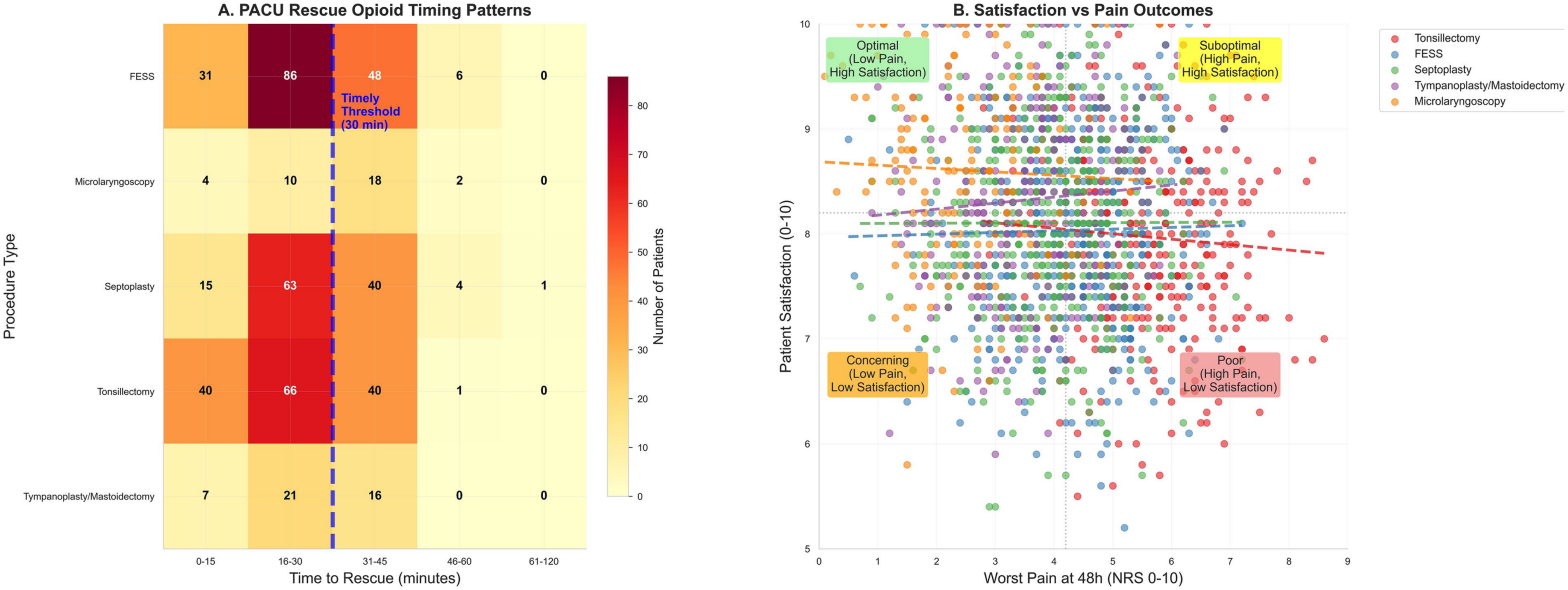
PACU management patterns and patient-reported outcomes. **(A)** Heat map of time to first rescue opioid in PACU among rescued patients, binned (0–15, 16–30, 31–45, 46–60, 61–120 min) by procedure; the dashed line at 30 min marks the severe-pain timeliness threshold, and the non-severe threshold is 45 min. **(B)** Satisfaction with pain management (0–10) versus worst pain at 48 h (NRS); scatter with locally weighted regression and 95% band.

Satisfaction was assessed using a single-item NRS (0–10, higher is better) administered by telephone or mobile app. This measure has face validity and has been used in prior perioperative cohort studies but lacks formal psychometric validation in the Chinese ENT surgery context and does not capture multidimensional recovery aspects such as functional restoration (return to oral intake, ambulation), sleep quality, anxiety, or interference with daily activities. Future studies should incorporate validated, domain-specific patient-reported outcome measures (e.g., Brief Pain Inventory interference subscale, Quality of Recovery-15, or procedure-specific instruments) to comprehensively evaluate patient experience and distinguish pain intensity from pain-related disability and emotional distress. The association between worst pain and satisfaction (Figure 1B) is consistent with prior literature but should be interpreted as descriptive rather than implying analgesia alone determines patient-centered quality.

#### 2.3.7 Outcomes

Two primary endpoints were prespecified. Primary endpoint A (patient-centered) was the worst pain at 48 h (continuous NRS). Primary endpoint B (process quality indicator) was a binary composite reflecting adequate pain care: receipt of scheduled non-opioid within 6 h AND, if severe PACU pain (NRS ≥ 7) occurred, rescue analgesia within 30 min. This audits protocol adherence rather than being a traditional patient outcome. When severe pain was absent, rescue was not required. If severe pain occurred without rescue, the composite was not achieved. Secondary endpoints were opioid consumption 0–24 h (OME, mg), PACU pain scores at arrival and discharge (NRS), PACU length of stay (minutes), PONV within 24 h, disposition (discharge vs. admission), 72-h unplanned contact, satisfaction (0–10), and adherence to scheduled non-opioids (among those prescribed).

### 2.4 Statistical analysis

Analyses followed STROBE guidance with procedure category (tonsillectomy, septoplasty, FESS, tympanoplasty/mastoidectomy, microlaryngoscopy; FESS reference) as the primary exposure for quantifying procedure-specific pain gradients, and secondary exposures including perioperative processes (intraoperative acetaminophen, NSAID, dexamethasone, local infiltration, discharge non-opioid scheduling) and patient characteristics (age, BMI, baseline pain, preoperative opioid status, comorbidities). Continuous variables were summarized as mean (SD) or median (IQR) and categorical variables as No. (%), with group comparisons using Kruskal-Wallis (continuous) and χ^2^ tests (categorical) at *α* = 0.05 two-sided; false discovery rate was controlled at 5% (Benjamini-Hochberg) for secondary hypotheses. For primary endpoint A (worst pain at 48 h, continuous NRS), ordinary least squares regression incorporated restricted cubic splines (four degrees of freedom) for age, BMI, and operative duration, adjusting for smoking, alcohol use, ASA status, OSA, diabetes, CKD/PUD, asthma/COPD, baseline pain, preoperative opioid status, procedure category, blood loss, local infiltration, intraoperative acetaminophen/NSAID/dexamethasone, antiemetic prophylaxis, weekend surgery, start time, order set use, residential setting, and surgeon/anesthesiologist fixed effects (anonymized codes) to control provider-level confounding, with heteroskedasticity-robust (HC3) standard errors for confidence intervals and *p*-values. Primary endpoint B (adequate pain care, binary composite) used logistic regression with identical covariates and splines, restricted to predictors occurring prior to or contemporaneous with the composite window to preserve temporal ordering, with adjusted odds ratios estimated using HC3 robust standard errors.

To address potential temporal bias over the 4.2-year accrual period, sensitivity analyses added calendar month (1-12) and year (2021–2025) as categorical indicators to all models, assessing stability of procedure-level pain gradients, process patterns, and key associations; model diagnostics included R^2^ and residuals (OLS) and AUC with calibration intercept/slope (logistic). Type I error adjustment for co-primary outcomes was not prespecified (limitation), though post-hoc inspection showed key findings (procedure pain differences *p* < 0.001; process predictors *p* < 0.01) remained significant at *α* = 0.025 without formal family-wise error control. Inverse-probability weighting assessed residual confounding using stabilized propensity scores (all covariates, procedure category, provider codes), with weights truncated at 1st/99th percentiles and covariate balance evaluated via standardized mean differences (<0.10 threshold). Primary outcomes and key covariates had no missing values, justifying complete-case analysis with data quality checks comprising range validations (NRS 0–10, non-negative times/OME), timestamp cross-verification, and medication-to-OME reconciliation using the institutional conversion table. Sample size reflected consecutive 4.2-year accrual (*N* = 1,632); *a priori* power calculations assumed SD ≈ 1.5 for 48 h pain and minimum subgroup *n* = 150, yielding ≥80% power for 0.5-NRS-unit differences (*α* = 0.05, two-sided), with observed median differences (1.0–3.1 units; section 3.5) exceeding this threshold and the process composite attainment rate (66.4%) yielding 95% CI half-width ±2.3 percentage points for precise estimation. Analyses used open-source software (Python: pandas, statsmodels, patsy, matplotlib) with version-controlled scripts.

## 3 Results

### 3.1 Cohort characteristics

The cohort comprised 1,632 adults, with a mean age of 38.5 years (SD 11.6) and a mean body mass index of 23.3 kg/m^2^ (SD 3.2); 901 (55.2%) were male. Marital status was predominantly married [1,171 (71.8%)]; education was secondary in 956 (58.6%) and tertiary in 495 (30.3%); employment was recorded as employed in 949 (58.2%) and student in 299 (18.3%). The residential distribution was urban in 1004 (61.5%), peri-urban in 300 (18.4%), and rural in 328 (20.1%), with public insurance in 1339 (82.1%). Smoking status was never in 1013 (62.1%), former in 260 (15.9%), and current in 359 (22.0%); hazardous alcohol use was present in 180 (11.0%). Caregiver accompaniment occurred in 1271 (77.9%), smartphone access in 1553 (95.2%), and interpreter needs in 37 (2.3%). ASA physical status was I in 626 (38.4%), II in 840 (51.5%), and III in 166 (10.2%), with diagnosed obstructive OSA in 111 (6.8%), diabetes in 130 (8.0%), CKD/PUD in 85 (5.2%), and asthma/COPD in 97 (5.9%). Preoperative opioid status was: no prior opioid use in 1408 (86.3%), intermittent use in 195 (11.9%), and chronic use in 29 (1.8%); regular preoperative non-opioid use occurred in 353 (21.6%). Baseline pain differed across procedures—highest for FESS [median, 4.1 (IQR, 3.2–5.2)] and lowest for micro-laryngoscopy [1.5 (0.8–2.3)], as shown in Table 1.

**Table 1:** Baseline sociodemographic and clinical characteristics by procedure category.

Characteristic	Overall (*N* = 1,632)	Tonsillectomy (*n* = 357)	Septoplasty (*n* = 423)	FESS (*n* = 489)	Tympanoplasty/Mastoidectomy (*n* = 214)	Microlaryngoscopy (*n* = 149)	*p*-value
Age, y	38.5 (11.6)	35.5 (10.8)	36.6 (11.7)	40.1 (11.1)	42.3 (10.6)	41.0 (11.8)	<0.001
BMI, kg/m^2^	23.3 (3.2)	22.9 (3.1)	23.1 (3.2)	23.5 (3.3)	23.6 (3.2)	23.4 (3.4)	<0.001
Travel time to hospital, min	40.0 (25.0–60.0)	40.0 (25.0–60.0)	35.0 (25.0–55.0)	45.0 (30.0–65.0)	45.0 (30.0–65.0)	35.0 (25.0–55.0)	<0.001
Household size, persons	3.0 (3.0–4.0)	3.0 (3.0–4.0)	3.0 (3.0–4.0)	3.0 (3.0–4.0)	3.0 (3.0–4.0)	3.0 (3.0–4.0)	0.694
Baseline pain (NRS 0–10)	3.2 (2.2–4.3)	2.2 (1.3–3.1)	3.5 (2.5–4.6)	4.1 (3.2–5.2)	2.0 (1.1–3.0)	1.5 (0.8–2.3)	<0.001
Sex							
Male	901 (55.2)	197 (55.2)	228 (53.9)	268 (54.8)	122 (57.0)	86 (57.7)	0.862
Female	731 (44.8)	160 (44.8)	195 (46.1)	221 (45.2)	92 (43.0)	63 (42.3)	
Marital status							
Single	378 (23.2)	118 (33.1)	127 (30.0)	76 (15.5)	25 (11.7)	32 (21.5)	<0.001
Married	1,171 (71.8)	224 (62.7)	282 (66.7)	378 (77.3)	170 (79.4)	117 (78.5)	
Divorced/widowed	83 (5.1)	15 (4.2)	14 (3.3)	35 (7.2)	19 (8.9)	0 (0.0)	
Education level							
Primary or less	181 (11.1)	28 (7.8)	32 (7.6)	88 (18.0)	22 (10.3)	11 (7.4)	<0.001
Secondary	956 (58.6)	216 (60.5)	260 (61.5)	264 (54.0)	132 (61.7)	84 (56.4)	
Tertiary	495 (30.3)	113 (31.7)	131 (31.0)	137 (28.0)	60 (28.0)	54 (36.2)	
Employment status							
Employed	949 (58.2)	186 (52.1)	257 (60.8)	284 (58.1)	132 (61.7)	90 (60.4)	<0.001
Unemployed	167 (10.2)	34 (9.5)	45 (10.6)	49 (10.0)	24 (11.2)	15 (10.1)	
Student	299 (18.3)	104 (29.1)	90 (21.3)	60 (12.3)	22 (10.3)	23 (15.4)	
Retired	217 (13.3)	33 (9.2)	31 (7.3)	96 (19.6)	36 (16.8)	21 (14.1)	
Residential setting							
Urban	1,004 (61.5)	214 (59.9)	274 (64.8)	284 (58.1)	136 (63.6)	96 (64.4)	0.118
Peri-urban	300 (18.4)	77 (21.6)	64 (15.1)	102 (20.9)	37 (17.3)	20 (13.4)	
Rural	328 (20.1)	66 (18.5)	85 (20.1)	103 (21.1)	41 (19.2)	33 (22.1)	
Insurance type							
Public	1,339 (82.1)	298 (83.5)	348 (82.3)	402 (82.2)	177 (82.7)	114 (76.5)	0.373
Private	123 (7.5)	21 (5.9)	36 (8.5)	35 (7.2)	16 (7.5)	15 (10.1)	
Self-pay	170 (10.4)	38 (10.6)	39 (9.2)	52 (10.6)	21 (9.8)	20 (13.4)	
Smoking status							
Never	1,013 (62.1)	232 (65.0)	262 (61.9)	304 (62.2)	129 (60.3)	86 (57.7)	0.641
Former	260 (15.9)	53 (14.8)	70 (16.5)	79 (16.2)	37 (17.3)	21 (14.1)	
Current	359 (22.0)	72 (20.2)	91 (21.5)	106 (21.7)	48 (22.4)	42 (28.2)	
Hazardous alcohol use							
Yes	180 (11.0)	34 (9.5)	48 (11.3)	61 (12.5)	23 (10.7)	14 (9.4)	0.658
No	1,452 (89.0)	323 (90.5)	375 (88.7)	428 (87.5)	191 (89.3)	135 (90.6)	
Caregiver accompaniment							
Yes	1,271 (77.9)	279 (78.2)	323 (76.4)	393 (80.4)	164 (76.6)	112 (75.2)	0.428
No	361 (22.1)	78 (21.8)	100 (23.6)	96 (19.6)	50 (23.4)	37 (24.8)	
Smartphone access							
Yes	1,553 (95.2)	341 (95.5)	404 (95.5)	456 (93.3)	206 (96.3)	146 (98.0)	0.069
No	79 (4.8)	16 (4.5)	19 (4.5)	33 (6.7)	8 (3.7)	3 (2.0)	
Interpreter needed							
Yes	37 (2.3)	7 (2.0)	10 (2.4)	14 (2.9)	3 (1.4)	3 (2.0)	0.799
No	1,595 (97.7)	350 (98.0)	413 (97.6)	475 (97.1)	211 (98.6)	146 (98.0)	
ASA physical status							
I	626 (38.4)	160 (44.8)	170 (40.2)	156 (31.9)	78 (36.4)	62 (41.6)	<0.001
II	840 (51.5)	171 (47.9)	214 (50.6)	290 (59.3)	107 (50.0)	58 (38.9)	
III	166 (10.2)	26 (7.3)	39 (9.2)	43 (8.8)	29 (13.6)	29 (19.5)	
OSA diagnosed							
Yes	111 (6.8)	24 (6.7)	30 (7.1)	33 (6.7)	13 (6.1)	11 (7.4)	0.994
No	1,521 (93.2)	333 (93.3)	393 (92.9)	456 (93.3)	201 (93.9)	138 (92.6)	
Diabetes							
Yes	130 (8.0)	23 (6.4)	35 (8.3)	45 (9.2)	18 (8.4)	9 (6.0)	0.548
No	1,502 (92.0)	334 (93.6)	388 (91.7)	444 (90.8)	196 (91.6)	140 (94.0)	
CKD or peptic ulcer disease							
Yes	85 (5.2)	19 (5.3)	22 (5.2)	29 (5.9)	9 (4.2)	6 (4.0)	0.808
No	1,547 (94.8)	338 (94.7)	401 (94.8)	460 (94.1)	205 (95.8)	143 (96.0)	
Asthma or COPD							
Yes	97 (5.9)	17 (4.8)	29 (6.9)	28 (5.7)	12 (5.6)	11 (7.4)	0.657
No	1,535 (94.1)	340 (95.2)	394 (93.1)	461 (94.3)	202 (94.4)	138 (92.6)	
NSAID allergy							
Yes	31 (1.9)	8 (2.2)	10 (2.4)	10 (2.0)	2 (0.9)	1 (0.7)	0.77
No	1,601 (98.1)	349 (97.8)	413 (97.6)	479 (98.0)	212 (99.1)	148 (99.3)	
Acetaminophen allergy							
Yes	15 (0.9)	2 (0.6)	5 (1.2)	5 (1.0)	2 (0.9)	1 (0.7)	0.966
No	1,617 (99.1)	355 (99.4)	418 (98.8)	484 (99.0)	212 (99.1)	148 (99.3)	
Opioid allergy							
Yes	19 (1.2)	3 (0.8)	5 (1.2)	6 (1.2)	3 (1.4)	2 (1.3)	0.986
No	1,613 (98.8)	354 (99.2)	418 (98.8)	483 (98.8)	211 (98.6)	147 (98.7)	
Preoperative opioid status							
Naive	1,408 (86.3)	314 (87.9)	365 (86.3)	422 (86.3)	188 (87.9)	119 (79.9)	0.231
Intermittent	195 (11.9)	37 (10.4)	52 (12.3)	60 (12.3)	24 (11.2)	22 (14.8)	
Chronic	29 (1.8)	6 (1.7)	6 (1.4)	7 (1.4)	2 (0.9)	8 (5.4)	
Regular preoperative non-opioid use							
Yes	353 (21.6)	78 (21.8)	86 (20.3)	111 (22.7)	48 (22.4)	30 (20.1)	0.784
No	1,279 (78.4)	279 (78.2)	337 (79.7)	378 (77.3)	166 (77.6)	119 (79.9)	

### 3.2 Operative and anesthetic characteristics

Median operative duration was 75 min (IQR, 50–105), ranging from 35 min for micro-laryngoscopy to 120 min for tympanoplasty/mastoidectomy; estimated blood loss was 70 mL (IQR, 40–120), lowest for micro-laryngoscopy [25 mL (18–32)] and highest for FESS (110 mL [60–180]). Intraoperative opioid exposure was 10.5 mg OME (IQR, 7.1–14.1). Laterality was midline/NA in 929 (56.9%), unilateral in 195 (12.0%), and bilateral in 508 (31.1%). Within tonsillectomy, cold steel was used in 193 (54.1%) and electrocautery in 164 (45.9%); within FESS, extent was limited in 303 (62.0%) and extensive in 186 (38.0%); within tympanoplasty, grafts were fascia in 153 (71.5%) and cartilage in 61 (28.5%); within microlaryngoscopy, laser (CO₂ or KTP) was used in 93 (62.4%) and cold instruments in 56 (37.6%). Local anesthetic infiltration was administered in 1187 (72.8%); maintenance used volatile agents in 1182 (72.4%) or TIVA in 450 (27.6%); airway devices were endotracheal tube in 1011 (62.0%) and laryngeal mask airway in 621 (38.1%). Intraoperative acetaminophen, NSAID, and dexamethasone were given in 1208 (74.0%), 1,011 (62.0%), and 1,337 (81.9%), respectively; ketorolac in 184 (11.3%), ketamine in 156 (9.6%), dexmedetomidine in 370 (22.7%), and antiemetic prophylaxis in 1241 (76.1%), as shown in Table 2.

**Table 2:** Operative and anesthetic characteristics by procedure category.

Characteristic	Overall (*N* = 1,632)	Tonsillectomy (*n* = 357)	Septoplasty (*n* = 423)	FESS (*n* = 489)	Tympanoplasty/Mastoidectomy (*n* = 214)	Microlaryngoscopy (*n* = 149)	*p*-value
Operative duration, min	75.0 (50.0–105.0)	45.0 (36.0–54.0)	55.0 (44.0–66.0)	93.0 (74.0–118.0)	120.0 (100.0–142.0)	35.0 (27.0–43.0)	<0.001
Estimated blood loss, mL	70.0 (40.0–120.0)	60.0 (40.0–80.0)	40.0 (25.0–55.0)	110.0 (60.0–180.0)	90.0 (55.0–125.0)	25.0 (18.0–32.0)	<0.001
Intraoperative opioid dose, OME mg	10.5 (7.1–14.1)	10.0 (6.9–13.3)	8.0 (5.4–10.7)	12.2 (8.6–16.2)	14.0 (9.8–18.7)	5.2 (3.3–7.1)	<0.001
Laterality							
Midline/NA	929 (56.9)	357 (100.0)	423 (100.0)	0 (0.0)	0 (0.0)	149 (100.0)	<0.001
Unilateral	195 (12.0)	0 (0.0)	0 (0.0)	195 (39.9)	0 (0.0)	0 (0.0)	
Bilateral	508 (31.1)	0 (0.0)	0 (0.0)	294 (60.1)	0 (0.0)	0 (0.0)	
Tonsillectomy technique							
Cold steel	193 (12.0)	193 (54.1)	0 (0.0)	0 (0.0)	0 (0.0)	0 (0.0)	<0.001
Electrocautery	164 (10.1)	164 (45.9)	0 (0.0)	0 (0.0)	0 (0.0)	0 (0.0)	
NA	1,275 (78.1)	0 (0.0)	423 (100.0)	489 (100.0)	214 (100.0)	149 (100.0)	
FESS extent							
Limited	303 (18.6)	0 (0.0)	0 (0.0)	303 (62.0)	0 (0.0)	0 (0.0)	<0.001
Extensive	186 (11.4)	0 (0.0)	0 (0.0)	186 (38.0)	0 (0.0)	0 (0.0)	
NA	1,143 (70.0)	357 (100.0)	423 (100.0)	0 (0.0)	214 (100.0)	149 (100.0)	
Tympanoplasty graft type							
Fascia	153 (9.4)	0 (0.0)	0 (0.0)	0 (0.0)	153 (71.5)	0 (0.0)	<0.001
Cartilage	61 (3.7)	0 (0.0)	0 (0.0)	0 (0.0)	61 (28.5)	0 (0.0)	
NA	1,418 (86.9)	357 (100.0)	423 (100.0)	489 (100.0)	0 (0.0)	149 (100.0)	
Local anesthetic infiltration							
Yes	1,187 (72.8)	127 (35.6)	341 (80.6)	425 (86.9)	196 (91.6)	98 (65.8)	<0.001
No	445 (27.3)	230 (64.4)	82 (19.4)	64 (13.1)	18 (8.4)	51 (34.2)	
Maintenance technique							
Volatile	1,182 (72.4)	273 (76.5)	294 (69.5)	361 (73.8)	148 (69.2)	106 (71.1)	0.043
TIVA	450 (27.6)	84 (23.5)	129 (30.5)	128 (26.2)	66 (30.8)	43 (28.9)	
Airway device							
ETT	1,011 (62.0)	228 (63.9)	252 (59.6)	307 (62.8)	144 (67.3)	80 (53.7)	0.036
LMA	621 (38.1)	129 (36.1)	171 (40.4)	182 (37.2)	70 (32.7)	69 (46.3)	
Intraoperative acetaminophen							
Yes	1,208 (74.0)	273 (76.5)	308 (72.8)	352 (72.0)	164 (76.6)	111 (74.5)	0.406
No	424 (26.0)	84 (23.5)	115 (27.2)	137 (28.0)	50 (23.4)	38 (25.5)	
Intraoperative NSAID							
Yes	1,011 (62.0)	176 (49.3)	250 (59.1)	331 (67.7)	149 (69.6)	105 (70.5)	<0.001
No	621 (38.1)	181 (50.7)	173 (40.9)	158 (32.3)	65 (30.4)	44 (29.5)	
Intraoperative dexamethasone							
Yes	1,337 (81.9)	290 (81.2)	349 (82.5)	403 (82.4)	176 (82.2)	119 (79.9)	0.942
No	295 (18.1)	67 (18.8)	74 (17.5)	86 (17.6)	38 (17.8)	30 (20.1)	
Intraoperative ketorolac							
Yes	184 (11.3)	38 (10.6)	48 (11.3)	61 (12.5)	20 (9.3)	17 (11.4)	0.801
No	1,448 (88.7)	319 (89.4)	375 (88.7)	428 (87.5)	194 (90.7)	132 (88.6)	
Intraoperative ketamine							
Yes	156 (9.6)	39 (10.9)	41 (9.7)	49 (10.0)	17 (7.9)	10 (6.7)	0.679
No	1,476 (90.4)	318 (89.1)	382 (90.3)	440 (90.0)	197 (92.1)	139 (93.3)	
Intraoperative dexmedetomidine							
Yes	370 (22.7)	66 (18.5)	104 (24.6)	111 (22.7)	51 (23.8)	38 (25.5)	0.336
No	1,262 (77.3)	291 (81.5)	319 (75.4)	378 (77.3)	163 (76.2)	111 (74.5)	
Antiemetic prophylaxis							
Yes	1,241 (76.1)	269 (75.4)	324 (76.6)	368 (75.3)	161 (75.2)	119 (79.9)	0.794
No	391 (24.0)	88 (24.6)	99 (23.4)	121 (24.7)	53 (24.8)	30 (20.1)	

Data are median (IQR) unless otherwise indicated; categorical variables are No. (%). *p*-values are from Kruskal–Wallis tests (continuous) or χ^2^ tests (categorical). FESS, functional endoscopic sinus surgery; OME, oral morphine equivalent; IQR, interquartile range.

### 3.3 Post-anesthesia care and early postoperative outcomes

Pain at PACU arrival was 5.6 (IQR, 4.8–6.5) and decreased to 4.3 (3.5–5.1) at discharge; the highest values occurred after tonsillectomy and the lowest after micro-laryngoscopy. Severe pain (NRS ≥ 7) occurred in 512 (31.4%) overall—52.1% after tonsillectomy, 27.9% after septoplasty, 33.5% after FESS, 12.1% after tympanoplasty/mastoidectomy, and 12.1% after micro-laryngoscopy. Rescue opioids were administered in 694 patients (42.5%) overall—61.6, 30.0, 47.0, 28.5, and 37.6% for tonsillectomy, septoplasty, FESS, tympanoplasty/mastoidectomy, and micro-laryngoscopy, respectively—with a median time to first rescue of 24 min (IQR, 17–30); among those receiving rescue, 566/694 (81.5%) met the prespecified timeliness threshold (≤30 min if severe or ≤45 min otherwise), corresponding to 34.7% of the full cohort. Total opioid consumption at 0–24 h was 9.3 mg OME (IQR, 5.7–13.0). PACU length of stay was 85 min (IQR, 68–102) without material differences across procedures; postoperative nausea/vomiting occurred in 304 (18.6%), sedation assessments were documented in 1166 (71.4%), and 1,433 (87.8%) were discharged on the day of surgery, as shown in Table 3 and Figure 1A.

**Table 3:** Post-anesthesia care and early postoperative outcomes by procedure category.

Characteristic	Overall (*N* = 1,632)	Tonsillectomy (*n* = 357)	Septoplasty (*n* = 423)	FESS (*n* = 489)	Tympanoplasty/Mastoidectomy (*n* = 214)	Micro-laryngoscopy (*n* = 149)	*p*-value
PACU pain on arrival (NRS 0–10)	5.6 (4.8–6.5)	6.6 (5.8–7.5)	5.5 (4.7–6.3)	5.8 (5.0–6.7)	4.8 (4.0–5.6)	4.2 (3.4–4.9)	<0.001
PACU pain at discharge (NRS 0–10)	4.3 (3.5–5.1)	5.2 (4.4–6.0)	4.2 (3.5–4.9)	4.4 (3.6–5.2)	3.6 (2.9–4.3)	3.2 (2.5–3.9)	<0.001
PACU length of stay, min	85.0 (68.0–102.0)	85.0 (69.0–101.0)	85.0 (68.0–102.0)	86.0 (69.0–103.0)	85.0 (69.0–102.0)	84.0 (67.0–101.0)	0.848
Opioid consumption 0–24 h, OME mg	9.3 (5.7–13.0)	12.0 (8.3–16.1)	8.0 (5.0–11.1)	9.1 (5.6–12.6)	7.0 (4.3–9.7)	5.0 (3.0–6.9)	<0.001
Time to first rescue dose, min (among those receiving rescue)	24.0 (17.0–30.0)	22.0 (16.0–29.0)	25.0 (18.0–31.0)	24.0 (17.0–30.0)	25.0 (18.0–31.0)	26.0 (19.0–32.0)	<0.001
Severe pain (NRS≥7) in PACU							
Yes	512 (31.4)	186 (52.1)	118 (27.9)	164 (33.5)	26 (12.1)	18 (12.1)	<0.001
No	1,120 (68.6)	171 (47.9)	305 (72.1)	325 (66.5)	188 (87.9)	131 (87.9)	
Rescue opioid given in PACU							
Yes	694 (42.5)	220 (61.6)	127 (30.0)	230 (47.0)	61 (28.5)	56 (37.6)	<0.001
No	938 (57.5)	137 (38.4)	296 (70.0)	259 (53.0)	153 (71.5)	93 (62.4)	
Timely rescue among rescued patients (≤30 min if severe; ≤45 min otherwise)							
Yes	566(81.5%)	199 (90.5%)	199 (90.5%)	199 (90.5%)	193 (83.9%)	40 (65.6%)	<0.001
No	128 (18.5%)	21 (9.5%)	26 (20.5%)	37 (16.1%)	37 (16.1%)	21 (34.4%)	
Scheduled non-opioid started within 6 h							
Yes	1,116 (68.4)	193 (54.1)	272 (64.3)	389 (79.5)	186 (86.9)	76 (51.0)	<0.001
No	516 (31.6)	164 (45.9)	151 (35.7)	100 (20.5)	28 (13.1)	73 (49.0)	
Postoperative nausea/vomiting 0–24 h							
Yes	304 (18.6)	78 (21.8)	76 (18.0)	95 (19.4)	34 (15.9)	21 (14.1)	0.228
No	1,328 (81.4)	279 (78.2)	347 (82.0)	394 (80.6)	180 (84.1)	128 (85.9)	
Sedation documentation present							
Yes	1,166 (71.4)	255 (71.4)	307 (72.6)	341 (69.7)	153 (71.5)	110 (73.8)	0.807
No	466 (28.6)	102 (28.6)	116 (27.4)	148 (30.3)	61 (28.5)	39 (26.2)	
Disposition							
Ambulatory discharge	1,433 (87.8)	313 (87.7)	377 (89.1)	427 (87.3)	189 (88.3)	127 (85.2)	0.649
Admission	199 (12.2)	44 (12.3)	46 (10.9)	62 (12.7)	25 (11.7)	22 (14.8)	

Data are median (IQR) unless otherwise indicated; categorical variables are No. (%). Time to first rescue dose is reported among patients who received rescue opioid in PACU. p values are from Kruskal–Wallis tests (continuous) or χ^2^ tests (categorical). PACU, post-anesthesia care unit; NRS, numeric rating scale; OME, oral morphine equivalent; PONV, postoperative nausea and vomiting; IQR, interquartile range.

### 3.4 Patient-reported outcomes (24–48 h) and discharge analgesic strategy

**Figure 2:**
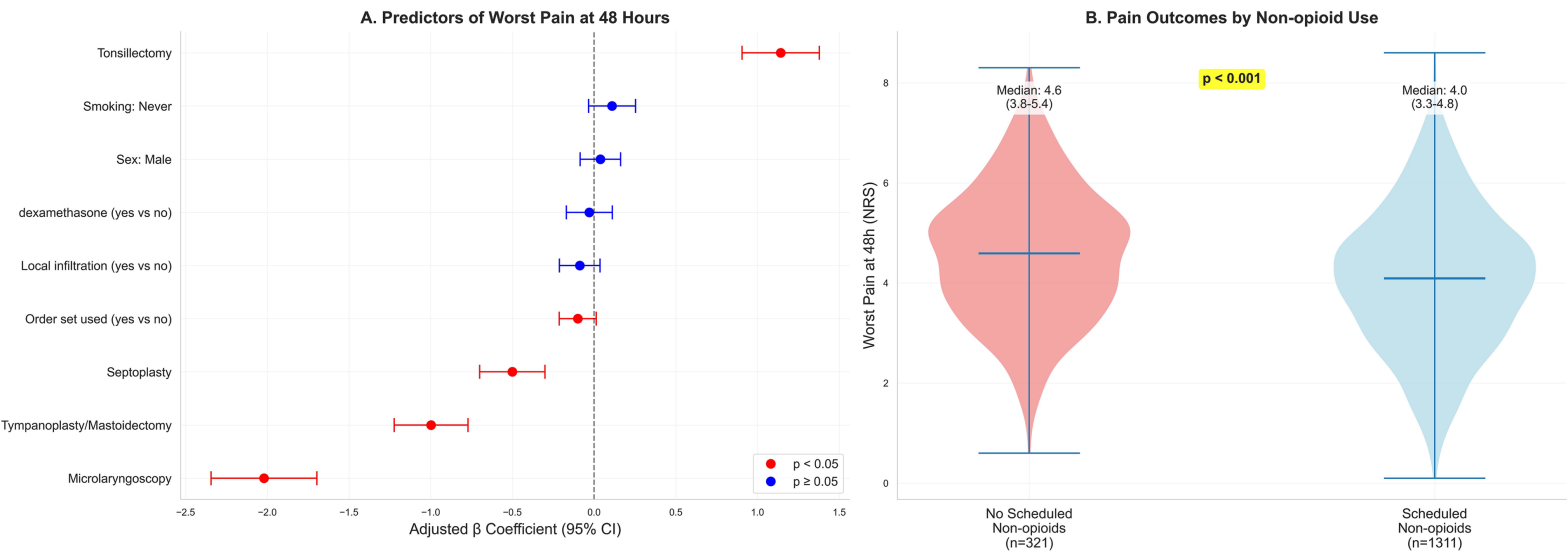
Determinants and distribution of 48-h pain. **(A)** Forest plot of adjusted *β* coefficients (95% CIs) from an ordinary least-squares model for worst pain at 48 h (continuous NRS), adjusted for prespecified demographics, comorbidities, baseline pain, perioperative processes, and provider fixed effects; restricted cubic splines for age, body mass index, and operative duration; heteroskedasticity-robust (HC3) standard errors. Positive β indicates higher pain. Predictors are ordered by absolute effect magnitude to enhance interpretability; procedure type (reference: FESS) emerged as the dominant determinant, with tonsillectomy associated with substantially higher 48-h pain (*β* = 1.14 units) and microlaryngoscopy with substantially lower pain (*β* = −2.02 units). Other perioperative processes showed smaller, often imprecise associations. **(B)** Violin/box plots of 48-h worst pain stratified by scheduled non-opioid at discharge (yes/no).

At discharge, 1311 (80.3%) received scheduled non-opioid analgesics and 359 (22.0%) received an opioid prescription. Patients with scheduled non-opioids had lower worst pain at 48 h (median, 4.0 vs. 4.6), lower 0–24-h opioid consumption (8.7 vs. 11.6 mg OME), fewer unplanned pain-related contacts within 72 h (8.9% vs. 14.3%), and higher satisfaction (mean, 8.3 vs. 7.9). Among those prescribed non-opioids, adherence at 48 h was 989 (75.4%), as shown in Table 4 and Figure 2B.

**Table 4:** Discharge prescribing and 48-h outcomes, stratified by scheduled non-opioids at discharge.

Characteristic	Overall (*N* = 1,632)	Non-opioids scheduled at discharge: Yes (*n* = 1,311)	No (*n* = 321)	*p*-value
Non-opioids scheduled at discharge	1,311 (80.3)	1,311 (100.0)	0 (0.0)	–
Opioid prescription at discharge	359 (22.0)	308 (23.5)	51 (15.9)	0.003
Unplanned pain-related contact within 72 h	162 (9.9)	116 (8.9)	46 (14.3)	0.004
Worst pain at 48 h (NRS 0–10)	4.2 (3.4–5.0)	4.0 (3.3–4.8)	4.6 (3.8–5.4)	<0.001
Satisfaction with pain management at 24–48 h (0–10)	8.2 (0.9)	8.3 (0.9)	7.9 (0.9)	<0.001
Opioid consumption 0–24 h, OME mg	9.3 (5.7–13.0)	8.7 (5.3–12.3)	11.6 (7.7–15.9)	<0.001
Adherence to scheduled non-opioids at 48 h (among those with scheduled non-opioids)	–	989 (75.4)	NA	–

Data are median (IQR) for pain and OME, mean (SD) for satisfaction, and No. (%) for categorical variables. p values compare patients discharged with vs without scheduled non-opioid analgesics using Mann–Whitney U tests (continuous) or χ^2^ tests (categorical). Adherence at 48 h is shown only among patients with scheduled non-opioids. OME, oral morphine equivalent; IQR, interquartile range. Adherence assessed only among patients discharged with scheduled non-opioids (*n* = 1,311): 989/1311 (75.4%).

### 3.5 Worst pain at 48 h by procedure category

Worst pain at 48 h (primary outcome A) varied substantially by procedure. Median (IQR) worst pain was: tonsillectomy 5.6 (4.8–6.4), FESS 4.5 (3.7–5.3), septoplasty 4.0 (3.2–4.8), tympanoplasty/mastoidectomy 3.5 (2.8–4.2), and microlaryngoscopy 2.5 (1.8–3.2) (*p* < 0.001, Kruskal-Wallis test). Mean (SD) values were: tonsillectomy 5.6 (1.2), FESS 4.5 (1.3), septoplasty 4.0 (1.4), tympanoplasty/mastoidectomy 3.6 (1.3), and microlaryngoscopy 2.5 (1.2). These unadjusted distributions are displayed in Figure 2B and summarized in Table 4.

### 3.6 Determinants of 48-h pain severity

In multivariable analysis, procedure category was the dominant determinant of worst pain at 48 h. Relative to FESS, tonsillectomy was associated with higher pain (*β* = 1.141; 95% CI, 0.905–1.378), whereas septoplasty (*β* = −0.501; 95% CI, −0.701 to −0.301), tympanoplasty/mastoidectomy (*β* = −0.998; 95% CI, −1.224 to −0.772), and micro-laryngoscopy (*β* = −2.021; 95% CI, −2.344 to −1.697) were associated with lower pain. Order set use showed a modest inverse association (*β* = −0.100; 95% CI, −0.213 to 0.013) that was not statistically precise, while other prespecified perioperative processes were not independently associated after adjustment, as shown in Table 5 and Figure 2A.

**Table 5:** Multivariable models for primary endpoints.

Predictor	Adjusted β (units)	95% CI (units)	*p*-value
Sex: Male	0.0381872	−0.086 to 0.162	0.5
Smoking status			
Former	0.0363371	−0.161 to 0.234	0.7
Never	0.109074	−0.034 to 0.253	0.13
ASA: II	−0.0186254	−0.140 to 0.103	0.76
ASA: III	−0.0435882	−0.232 to 0.145	0.65
Preop opioid: Intermittent	−0.260682	−0.751 to 0.229	0.29
Preop opioid: Naive	−0.272852	−0.736 to 0.191	0.21
Procedure type			
Microlaryngoscopy	−2.02082	−2.344 to −1.697	<0.001
Septoplasty	−0.501293	−0.701 to −0.301	<0.001
Tonsillectomy	1.14138	0.905 to 1.378	<0.001
Tympanoplasty/mastoidectomy	−0.998397	−1.224 to −0.772	<0.001
Residence			
Rural	−0.0390897	−0.219 to 0.141	0.6
Urban	0.0011504	−0.149 to 0.151	0.98
Baseline pain (per 1 NRS)	−0.0168549	−0.057 to 0.024	0.41
0–24 h opioid, OME (per 5 mg)	−0.0105097	−0.071 to 0.050	0.73
Local infiltration (yes vs. no)	−0.0880367	−0.212 to 0.036	0.14
Intraop NSAID (yes vs. no)	−0.0181638	−0.134 to 0.098	0.75
Intraop dexamethasone (yes vs. no)	−0.0297172	−0.170 to 0.111	0.67
Antiemetic prophylaxis (yes vs. no)	0.0816282	−0.051 to 0.214	0.22
Weekend surgery (yes vs. no)	0.0304512	−0.092 to 0.153	0.62
Order set used (yes vs. no)	−0.0999741	−0.213 to 0.013	0.08

Primary endpoint A—worst pain at 48 h (NRS, continuous). Model: ordinary least squares with restricted cubic splines for age, BMI, and operative duration; provider fixed effects for surgeon and anesthesiologist; heteroskedasticity-robust (HC3) SEs. Reference categories: Female sex; ASA I; Preoperative opioid status—Chronic; Procedure—FESS; Smoking—Current; Residential setting—Peri-urban. Positive β indicates higher worst pain at 48 h.

### 3.7 Reliability of acute analgesia processes and care quality

**Figure 3:**
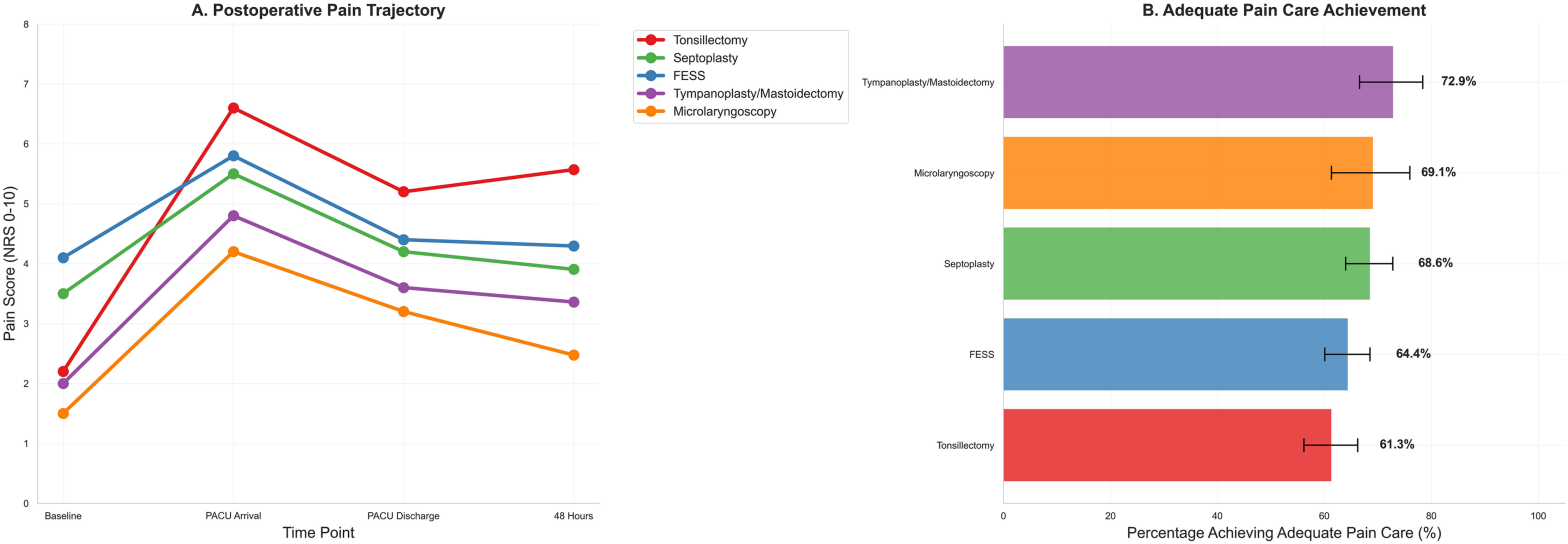
Postoperative pain trajectory and adequate pain care by procedure. **(A)** Postoperative pain trajectory from baseline through PACU arrival, PACU discharge, and 48 h, stratified by procedure. **(B)** Adequate pain care composite—scheduled non-opioid within 6 h plus timely rescue if severe PACU pain occurred (rescue not required if no severe pain); bars show proportions with 95% confidence intervals across procedure categories.

The composite of adequate pain care was achieved in 66.4% overall, with procedure-level achievement ranging from 61.3% after tonsillectomy to 72.9% after tympanoplasty/mastoidectomy (Figure 3B). This variation likely reflects differential implementation of standardized protocols rather than intentional inequity, with tonsillectomy cases experiencing higher pain severity necessitating more complex rescue logistics. Sensitivity analyses incorporating calendar month and calendar year indicators to adjust for potential temporal changes in practice patterns, medication availability, and care protocols over the 4.2-year study period did not materially alter point estimates or confidence intervals for procedure-level pain effects (maximum change in *β* for any procedure: 0.08 units), process composite associations (maximum change in OR: 0.03), or other key predictors, suggesting that primary findings were robust to secular trends. Nevertheless, unmeasured practice evolution (e.g., gradual adoption of specific protocols, shifts in opioid prescribing culture) cannot be fully excluded. In the process-centered model, higher 0–24-h opioid exposure was inversely associated with achieving the composite (adjusted OR per 5 mg OME, 0.921; 95% CI, 0.898–0.944), intraoperative NSAID use was positively associated (OR, 1.254; 95% CI, 1.000–1.573), and dexamethasone was inversely associated (OR, 0.679; 95% CI, 0.508–0.907). Procedure-level achievement of the composite ranged from 61.3% after tonsillectomy to 72.9% after tympanoplasty/mastoidectomy, as shown in Table 6 and Figure 3B.

**Table 6:** Primary endpoint B—adequate pain care (binary composite)^†^.

Predictor	Adjusted OR	95% CI	*p*-value
Baseline pain (per 1 NRS)	1.02391	0.948–1.106	0.549
Local infiltration (yes vs. no)	0.949218	0.735–1.225	0.689
0–24 h opioid, OME (per 5 mg)	0.920673	0.898–0.944	<0.001
Intraop NSAID (yes vs. no)	1.25398	1.000–1.573	0.040
Intraop dexamethasone (yes vs. no)	0.678918	0.508–0.907	<0.001
Antiemetic prophylaxis (yes vs. no)	1.16763	0.903–1.510	0.238
Weekend surgery (yes vs. no)	1.20641	0.948–1.536	0.128
Order set used (yes vs. no)	1.0171	0.810 to 1.278	0.884

Model: logistic regression with restricted cubic splines for age, BMI, and operative duration; provider fixed effects (surgeon and anesthesiologist); heteroskedasticity-robust (HC3) SEs. Reference categories as in Table 5. ^†^Adequate pain care was defined as receipt of a scheduled non-opioid within 6 h postoperatively and timely rescue [≤30 min if severe PACU pain (NRS ≥ 7); ≤45 min otherwise]; for patients without severe PACU pain, the rescue criterion was not invoked.

### 3.8 Multivariable structure and patient-reported experience

**Figure 4:**
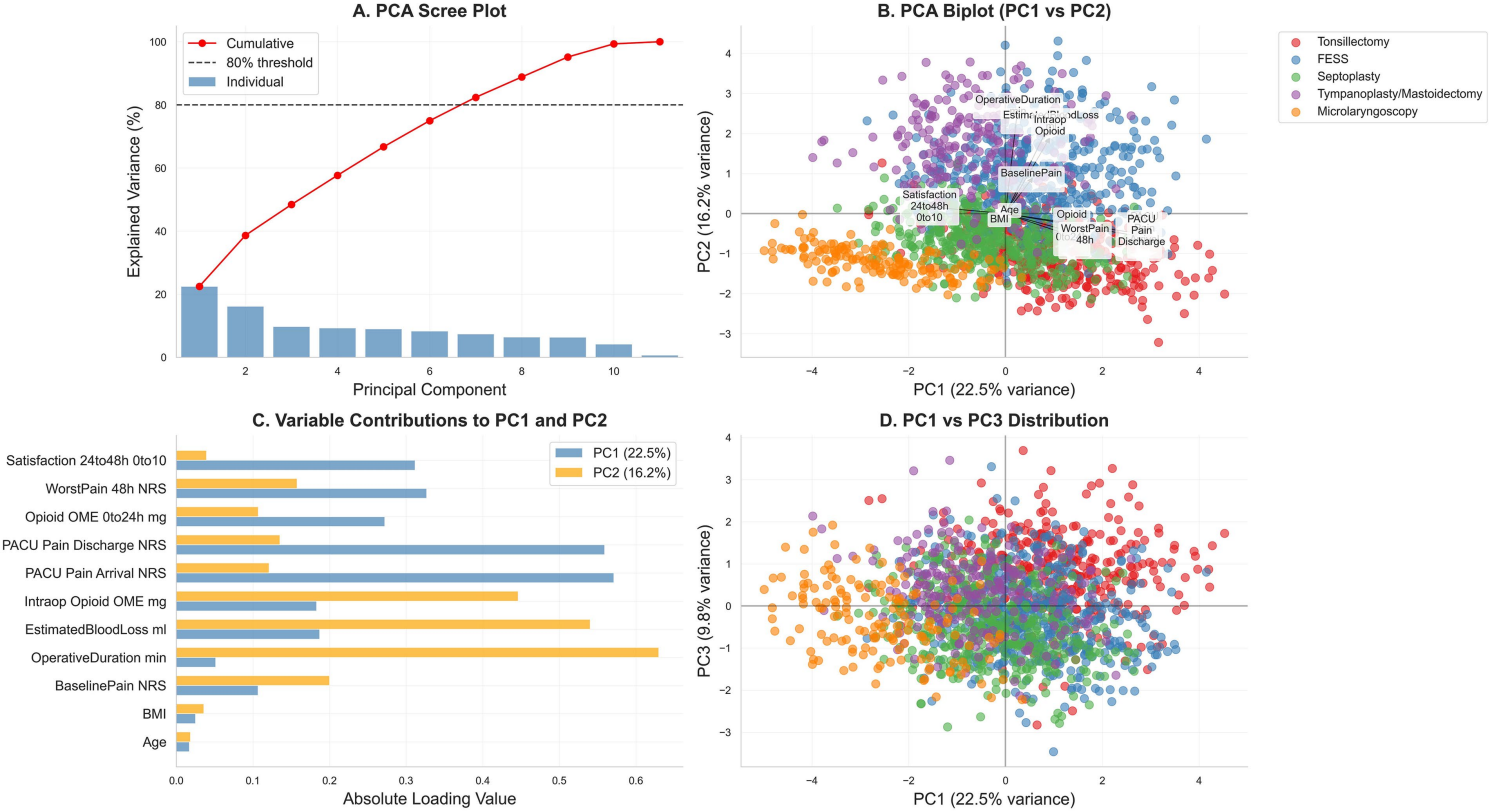
Principal-component analysis (PCA) of centered, z-score–standardized perioperative variables. **(A)** Scree plot with cumulative variance (dotted line marks 80%). **(B)** PC1–PC2 biplot showing variable loadings (arrows) and observations colored by procedure (tonsillectomy, FESS, septoplasty, tympanoplasty/mastoidectomy, microlaryngoscopy). **(C)** Absolute loadings for PC1 and PC2. **(D)** PC1–PC3 score distribution by procedure. Variables include age, BMI, baseline pain, PACU pain (arrival, discharge), worst pain at 48 h, satisfaction at 24–48 h, operative duration, estimated blood loss, and opioid exposure (intraoperative, PACU, total 0–24-h oral morphine equivalents); axes display percent variance explained.

Principal component analysis confirmed construct validity: pain indices and opioid exposure loaded strongly on PC1 (22.5% variance), distinct from operative characteristics on PC2 (16.2% variance). Procedure-specific clustering in PC1-PC2 space (Figures 4B,D) validated that the five procedure categories represent genuinely distinct patient populations with different pain profiles, supporting procedure-stratified analysis. Variable loadings (Figure 4C) were consistent with hypothesized relationships. Patient satisfaction correlated inversely with 48-h pain (Figure 1B), confirming pain intensity as a key driver of patient experience.

## 4 Discussion

This single-center cohort characterizes contemporary perioperative pain care across five common otolaryngology procedures in China and demonstrates clinically meaningful heterogeneity in nociceptive burden, process reliability, and downstream patient-reported outcomes. As hypothesized, procedure category was the dominant determinant of 48-h pain severity, with tonsillectomy yielding substantially higher pain than other procedures and microlaryngoscopy the lowest, supporting procedure-tailored analgesic pathways. The pattern of highest pain intensity after tonsillectomy and lowest after micro-laryngoscopy aligns with procedure-specific literature, reinforcing the importance of tailored multimodal pathways in otolaryngology practice (5, 11, 27).

The procedure-stratified gradients in baseline pain and intraoperative characteristics are concordant with prior reports. Septoplasty and rhinoplasty typically yield mild, self-limited pain trajectories, whereas FESS produces predominantly mild-to-moderate pain, and micro-laryngoscopy-related throat discomfort is linked more to suspension duration than to applied pressure, collectively mirroring our observed distribution (5, 11, 28). For tonsillectomy, international guidance advocates a non-opioid foundation combining acetaminophen and NSAIDs with a single intraoperative dose of dexamethasone, reserving opioids for rescue, which is consistent with the high nociceptive burden we observed and the observed benefits of non-opioid scheduling post-discharge (28, 29).

The prevalence of severe pain on PACU arrival or during recovery in this cohort (operationalized as NRS ≥ 7) is broadly consistent with global PACU literature in which 30–50% of patients experience moderate-to-severe pain despite contemporary practices (3, 30, 31). The median time to first rescue of 24 min and the finding that 81.5% of rescued patients met the prespecified timeliness threshold (34.7% of the overall cohort) highlights opportunities to improve response reliability, echoing calls to treat timeliness as a quality indicator in acute pain care (3, 32). Procedure-level variation in severe pain and rescue rates—most pronounced after tonsillectomy—further supports procedure-specific staffing prompts and standing orders in high-risk lists (3, 26, 30).

Post-discharge outcomes in this cohort suggest that routine scheduling of non-opioid analgesics was associated with lower worst pain at 48 h; however, residual confounding by indication cannot be excluded, lower early opioid exposure, fewer unplanned pain-related contacts, and higher satisfaction, which is directionally consistent with ERAS-style, opioid-sparing paradigms and primary care guidance for ambulatory surgery (19, 33, 34). These associations are biologically plausible, given the well-established opioid-sparing effect of NSAIDs and acetaminophen in surgical populations and the absence of a clinically important increase in bleeding with perioperative NSAIDs in contemporary meta-analysis (33–35). In head and neck ambulatory surgery specifically, emerging evidence indicates that ibuprofen can be permitted and that default opioid quantities after ESS can be substantially reduced without compromising pain control, which coheres with the present pattern of lower 0–24-h OME when non-opioids are structured (35, 36).

Multivariable modeling identified procedure category as the dominant determinant of 48-h pain severity, a finding congruent with procedure-specific guidance and observational data that show tonsillectomy as an outlier for intensity and duration of pain (5, 11, 27, 34). The modest inverse association of order-set use with pain severity, while imprecise, is consistent with literature linking pathway compliance to improved perioperative outcomes in head and neck surgery, though evidence remains limited and mostly observational (34, 37). These inverse associations likely reflect confounding by indication and temporal ordering rather than causal decrements in process quality. Notably, dexamethasone showed an inverse association with the composite process measure in this cohort, which contrasts with guidance recommending intraoperative dexamethasone for its antiemetic and modest analgesic benefits; this discrepancy likely reflects confounding by indication (e.g., selective administration in anticipated higher-risk cases) and underscores the need for caution when interpreting process–outcome relations in nonrandomized settings (37–39).

The process analysis also found that greater 0–24-h opioid exposure correlated with lower probability of achieving the composite “adequate pain care,” which may indicate reactive prescribing in response to poorly controlled pain rather than a causal decrement in process quality; nonetheless, the directionality is consistent with opioid-sparing ERAS elements and international comparisons showing markedly lower opioid exposure when non-opioid-first pathways are embedded (19, 40). The positive association between intraoperative NSAID administration and process attainment is likewise aligned with meta-analytic evidence for NSAIDs as core opioid-sparing agents in perioperative care, including in otolaryngology (33, 35).

Exploratory multivariate structure (PCA) in this cohort suggested that pain indices and opioid exposure co-varied strongly, whereas operative duration and intraoperative features formed an orthogonal dimension, a pattern that is consistent with procedure-driven case-mix and known determinants of acute pain trajectories (36, 41, 42). The inverse association between patient-reported satisfaction and worst pain at 48 h is expected and coheres with patient-reported outcomes research in perioperative settings (36). The composite “adequate pain care” metric, while innovative, requires careful interpretation. It is a process quality indicator—not a traditional patient-centered outcome—and its components can function as both exposures (non-opioid prescribing and rescue timing as interventions) and outcomes (measures of protocol adherence). This dual nature may create conceptual ambiguity, but is consistent with quality improvement science, where process reliability is itself an endpoint. Future studies should disentangle the causal pathway by treating early non-opioid initiation and timely rescue as time-varying exposures in causal inference models (e.g., marginal structural models) to estimate their independent effects on pain, opioid use, and satisfaction.

In China, the national registry data indicate a substantial burden of moderate-to-severe postoperative pain and heterogeneous practice patterns, supporting the generalizability of our observations and the potential impact of standardizing non-opioid-first, time-sensitive rescue protocols (24). Cross-national comparisons further suggest that prescribing culture and institutional pathways strongly influence opioid exposure, implying that procedure-specific, ERAS-consistent protocols adapted to Chinese tertiary centers could reduce variability while maintaining analgesic effectiveness (12, 43, 44). The observed procedural variation in composite attainment (61.3% for tonsillectomy vs. 72.9% for tympanoplasty/mastoidectomy) primarily reflects local quality gaps in protocol implementation rather than generalized clinical insights. This finding serves as an internal audit signal for our institution to standardize care processes, particularly for high-pain procedures like tonsillectomy, but offers limited guidance for external readers beyond demonstrating feasibility of process metrics for quality monitoring.

In sum, this single-center analysis corroborates procedure-specific pain gradients in ENT surgery, demonstrates clinically and operationally meaningful associations between non-opioid-first strategies and improved early outcomes, and identifies timeliness of rescue as a modifiable process target. The alignment and occasional discordance with existing guidance—particularly around dexamethasone—emphasize the value of local audit with robust adjustment and, where feasible, targeted implementation trials to determine causality in the Chinese otolaryngology context (11, 27, 34, 43, 45).

This study enrolled a large consecutive single-center cohort over 4.2 years (*N* = 1,632) across five common otolaryngology procedures, with detailed procedural descriptors and comprehensive linkage of electronic health, anesthesia, medication, and prescribing records. Complete capture of primary outcomes and covariates minimized information bias, while time-stamped medication and pain assessments enabled objective measurement of rescue timing and process performance. The prespecified analytic plan employed contemporary methods—including restricted cubic splines, provider fixed effects, heteroskedasticity-robust standard errors, seasonality and secular trend adjustment, and inverse-probability weighting with balance diagnostics—supported by reproducible code and a single formulary for OME conversions.

However, there are also various limitations. First, the single-center observational design substantially constrains external validity; care processes, staffing models, and prescribing culture at this tertiary academic center in Guangzhou may not generalize to community hospitals, non-academic settings, or institutions with different ERAS implementation, formulary restrictions, or nurse-to-patient ratios. Multicenter validation is essential. The observational design precludes causal inference. Second, several important confounders were unmeasured: provider characteristics (surgeon/anesthesiologist seniority, experience) were controlled only via fixed-effects codes; PACU operational factors (real-time nurse-to-patient ratios, competing demands, nursing experience) were not documented; procedure-specific technical details (tonsillectomy dissection plane, granular electrocautery settings, suspension duration) were not uniformly recorded; anesthetic depth (processed EEG) and hemodynamic variability were not captured; and psychiatric comorbidities (anxiety, depression, PTSD, substance use disorders) were not systematically documented. These conditions influence pain perception, catastrophizing, and opioid response. Confounding by indication (notably dexamethasone) may reflect selective administration in higher-risk cases. Third, the composite thresholds (≤30 min for NRS ≥ 7; ≤45 min otherwise) are pragmatic but somewhat arbitrary; alternative definitions (e.g., 20 min, patient-reported time-to-relief) may be equally valid. The 30-min threshold, while based on prior literature and workflow realities, may be insufficiently stringent; more aggressive benchmarks (≤15 min) would better align with patient-centered principles but require workflow redesign and enhanced staffing. Fourth, satisfaction was assessed using a single-item NRS lacking formal psychometric validation and not capturing multidimensional recovery (functional restoration, sleep, anxiety). Pain intensity and adherence are patient-reported and subject to cultural biases. Fifth, complete-case analysis assumes data were missing completely at random. Sixth, longer-term outcomes (bleeding, chronic pain at 3–6 months, readmissions, opioid dependence) were not assessed. Seventh, although sensitivity analyses adjusting for calendar year/month suggested stability, unmeasured temporal changes in technique refinement, protocol adoption, and prescribing norms over 4.2 years cannot be excluded. Eighth, several sociodemographic variables (household size, travel time, smartphone access) showed minimal association with outcomes; their inclusion may appear excessive, though retained for comprehensive characterization. Ninth, formal Type I error adjustment for two co-primary outcomes was not prespecified, though major associations remained significant at *α* = 0.025. Finally, the study was not prospectively registered in a public trial registry, limiting transparency.

## 5 Conclusion

This single-center analysis demonstrates that postoperative nociceptive burden and early care reliability in common ENT procedures are chiefly determined by the operation performed, with tonsillectomy showing the highest pain intensity and micro-laryngoscopy the lowest. Two pragmatic levers—routine scheduling of non-opioid analgesics and timely rescue for severe PACU pain—were associated with lower 48-h pain, reduced early opioid exposure, fewer unplanned contacts, and improved satisfaction. In contrast, greater early opioid use tracked with poorer process attainment, underscoring the value of an opioid-sparing multimodal paradigm. These findings, derived from complete capture of primary outcomes and robust modeling with provider adjustment, provide internally valid estimates for a single Chinese tertiary academic center and support the adoption of standardized, procedure-specific pathways and auditable timeliness metrics at similar institutions, pending multicenter validation to establish external validity and generalizability across diverse practice environments. Future work should extend these observations through multicenter validation and prospective implementation studies to determine causal impact and scalability across diverse health-system contexts.

## Data Availability

The raw data supporting the conclusions of this article will be made available by the authors, without undue reservation.
